# The Population Health Model (POHEM): an overview of rationale, methods and applications

**DOI:** 10.1186/s12963-015-0057-x

**Published:** 2015-09-03

**Authors:** Deirdre A. Hennessy, William M. Flanagan, Peter Tanuseputro, Carol Bennett, Meltem Tuna, Jacek Kopec, Michael C. Wolfson, Douglas G. Manuel

**Affiliations:** Health Analysis Division, Statistics Canada, 100 Tunney’s Pasture Driveway, Ottawa, ON K1A 0T6 Canada; Ottawa Hospital Research Institute, Room 2-012 Administrative Services Building, Box 684, 1053 Carling Ave., Ottawa, ON K1Y 4E9 Canada; C.T. Lamont Primary Health Care Research Centre and Bruyere Research Institute, 43 Bruyere Street, Ottawa, ON K1N 5C8 Canada; The Institute for Clinical Evaluative Sciences, G1 06, 2075 Bayview Avenue, Toronto, ON M4N 3M5 Canada; School of Population and Public Health, University of British Columbia and the Arthritis Research Centre of Canada, 895 West 10th Avenue, Vancouver, BC V5Z 1L7 Canada; Faculty of Medicine, University of Ottawa, 451 Smyth Road, Ottawa, ON K1H 8M5 Canada; The Department of Family and Department of Epidemiology and Community Medicine, University of Ottawa, Room 3105, 451 Smyth Road, Ottawa, ON K1H 8M5 Canada

## Abstract

The POpulation HEalth Model (POHEM) is a health microsimulation model that was developed at Statistics Canada in the early 1990s. POHEM draws together rich multivariate data from a wide range of sources to simulate the lifecycle of the Canadian population, specifically focusing on aspects of health. The model dynamically simulates individuals’ disease states, risk factors, and health determinants, in order to describe and project health outcomes, including disease incidence, prevalence, life expectancy, health-adjusted life expectancy, quality of life, and healthcare costs. Additionally, POHEM was conceptualized and built with the ability to assess the impact of policy and program interventions, not limited to those taking place in the healthcare system, on the health status of Canadians. Internationally, POHEM and other microsimulation models have been used to inform clinical guidelines and health policies in relation to complex health and health system problems. This paper provides a high-level overview of the rationale, methodology, and applications of POHEM. Applications of POHEM to cardiovascular disease, physical activity, cancer, osteoarthritis, and neurological diseases are highlighted.

## Introduction

The POpulation HEalth Model (POHEM) was conceived and developed at Statistics Canada in the early 1990s. POHEM was built in response to concerns about a lack of data around population health status and health outcomes [1]. Additionally, the traditional focus of health policy on funding an increasingly expensive healthcare system was coming into question. Policymakers wanted a solid understanding of population health benefits and implications for resource allocation when making decisions regarding the comparative merits of a range of health interventions [1]. At that time, a new concept of population health was emerging in Canada as a result of the 1974 Lalonde report that identified determinants of health outside of the healthcare system [2]. Lalonde, and later Evans and Stoddart [3, 4] among others, called for a broader perspective on the determinants of health, emphasizing the role of the social environment. Pervasive evidence was then coming to light of the strong correlation between socioeconomic status and health, which created a growing appreciation that the impact of medical interventions and the healthcare system on health was much less than thought[4]. This perspective necessitated a corresponding broader set of health information, including data that captured health status, health outcomes, and the effect of health interventions [1].

A comprehensive system of health statistics was proposed [1, 5]. The system centered around the lifecycle of the individual, and incorporated the external environment (physical, social, and economic) as well as a range of interventions both individual (e.g. cholesterol-lowering medication) and collective (e.g. smoking bans). The POHEM mircosimulation model was the central part of this proposed system. POHEM allowed for the simulation of the lifecycle dynamics and the health status and health outcomes of the Canadian population by integrating multivariable data collected from a range of sources and by projecting hypothetical alternative population distributions of health outcomes where one or more factors could be changed based on an intervention [1]. POHEM was conceptualized and built with the ability to assess the impact of a wide range of policy and program interventions, not limited to those taking place in the healthcare system, on the health status of the Canadian population.

In population health, policy and program evaluation has traditionally been undertaken after implementation. This approach has a number of drawbacks. First, knowing that large-scale expensive programs can sometimes fail, policymakers may be reluctant to implement them despite their potential benefits. Second, failure or unforeseen consequences of the policy or program may only be revealed after pilot studies or full implementation. Along with other modeling methodologies, microsimulation modeling offers a method of evaluation that allows policymakers to examine the results, consequences, and benefits of a program in advance of implementation [6]. These methods, while typically used to evaluate programs outside healthcare (e.g., taxation and pension policy)[7], are becoming increasingly used in the health arena [6, 8–10]. Internationally, substantial progress has been made using microsimulation modeling to inform health policy, especially in the areas of cancer treatment, obesity, and chronic disease [11–14]. Beyond policy and program evaluation, microsimulation methods can also play an important role in population or public health planning. In Canada, agencies such as Statistics Canada, Health Canada, the Public Health Agency of Canada (PHAC), and the Canadian Partnership Against Cancer (CPAC) have all employed microsimulation models to project future incidence and prevalence of risk factors and diseases, and future demands for healthcare resources [15–24]. These projections are useful for planning and implementation of large scale public health programs, and can also constitute a “business case” for policymakers. POHEM is one such model.

The purpose of this paper is to provide a high-level overview of the rationale, methods and applications of POHEM. The following discussion of microsimulation and POHEM is largely non-technical. Interested readers are referred to the following citations for additional details [1, 7, 16, 18–28].

### Overview of methods

#### What is POHEM?

**Fig. 1:**
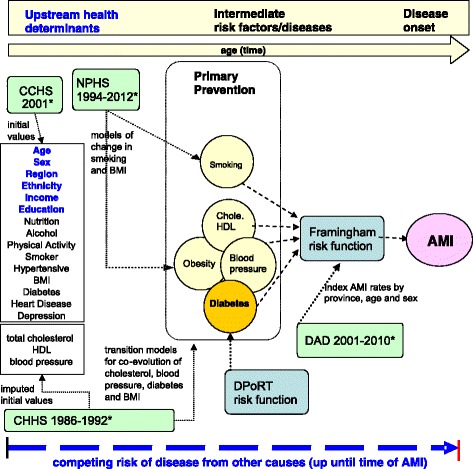
Schematic diagram of POHEM-cardiovascular disease (CVD) model. The schematic diagram of the POHEM-CVD model shows the variables, data sources (green boxes), risk factors (yellow circles) and risk algorithms (blue boxes) modelled to project cardiovascular outcomes (pink oval). Notes: * A detailed description of the data sources is available in Appendix. CHHS = Canadian Heart Health Study, NPHS = National Population Health Survey, CHHS = Canadian Community Health Survey, DAD = Discharge abstract database, BMI = body mass index, Chole = Cholesterol, HDL = high density lipoprotein, DPoRT = diabetes population risk tool, AMI = acute myocardial infarction

To describe POHEM, it is first necessary to define dynamic microsimulation. Dynamic microsimulation, in the context of social science and population health, is the simulation of large samples of individuals (micro) and their behaviours, states and actions, over time (dynamic), in order to project the socioeconomic and demographic developments of society [7]. By necessity, individuals’ behaviours, states and actions are modeled using multiple sources of empirical data, including cross-sectional surveys, longitudinal surveys, administrative databases, vital statistics, and Census data. See Fig. 1 for an illustration of the data sources used in the POHEM cardiovascular disease model and the Appendix for a more comprehensive description of each data source. The explicit microanalytic foundation of these models is important in representing realistic population heterogeneities and is conducive to simulating the effects of policy interventions.

POHEM is one of a few population-based health dynamic microsimulation models worldwide; Zucchelli *et al.* and others have reviewed the field [6, 8–10]. The model dynamically simulates individuals’ disease states, risk factors, and health determinants, in order to describe and project health outcomes, including disease incidence, prevalence, life expectancy, health-adjusted life expectancy, health-related quality of life, and healthcare costs. POHEM simulates discrete events, such as changes in disease states, on a case-by-case basis in continuous time. Events occur in chronological sequence at specific moments in time and the probability of an event occurring, or more precisely, the time to an event, is determined by a random process that draws from empirically observed and estimated waiting time distributions for each event. The occurrence of diverse events is embedded in a competing risk framework, that is, events compete with each other to be the next to occur. As events occur they influence subsequent draws from the relevant waiting time distributions, and hence the life trajectory of the individual. These many random components allow individuals’ life course trajectories to vary in ways that realistically reproduce observed patterns of behavior, including variation that is not “explained” by conventional epidemiological risk functions. The resulting variability in simulated individuals’ life course trajectories builds on the variations in the initial values assigned to the simulated individual. The resulting life trajectory of each simulated individual is influenced by exposure to simulated real world events, such as smoking initiation, changes in body mass index (BMI), and incidence of disease.

#### How does POHEM work?

**Fig. 2:**
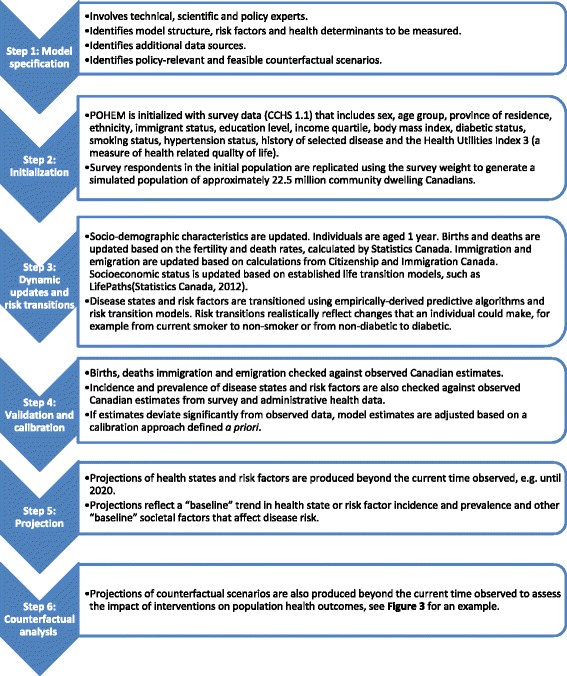
Process of producing estimates from a POHEM model. The figure presents a flow diagram to summarize the 6 step process of producing estimates from a POHEM model. Additional detail about each step of the process is provided in the body of the manuscript

Producing estimates from POHEM involves six steps, described below and in Fig. 2.

##### **Step 1- Model specification**

Model specification or conceptualization is a critical step, involving in-depth consultation among microsimulation experts, clinical and population health experts, and policymakers [29]. In this process, priority policy questions to be addressed, model functionalities (e.g. potential interventions and key outcome variables), and the policy-relevant and feasible counterfactual scenarios are decided.

##### **Step 2- Initialization**

POHEM can be initialized in two different ways. The first approach creates a synthetic population of Canadians. Synthetic populations are ones where the individual simulated lives are completely hypothetical. They are generated based on individual-level data, but the information extracted from these data mainly take the form of transition patterns among health and socioeconomic states – for example, fertility rates, patterns of smoking initiation, and risks of developing cancer or a heart attack. These simulations generate “cases”, i.e. individual synthetic biographies, starting at birth and moving through life event by event until death. These simulations calibrate historical births so that, when combined with mortality hazards, the age-sex structure of the population is complete and representative from 1971 to 2005, and a full population is projected until 2050 based on Statistics Canada’s official population projections [30]. An important advantage of simulating with a synthetic cohort is the coverage of individuals’ full life course, including risk exposures, from birth to death. The challenge is a lack of historical information about risk exposures, for example smoking.

The second approach creates a starting population from a cross-sectional survey of Canadians, weighted to reflect the whole population over the age of 20 years (approximately 22.5 million people). Most current POHEM models are initialized in 2001, and reflect the population base of the 2001 Canadian Community Health Survey 1.1 (CCHS). A key advantage of using the initial or start-up population directly from a survey is that it provides a wide set of individual characteristics where the multivariate joint distribution of these variables is empirically based, and draws on a large number of respondents (CCHS 1.1 *n* = 105,908) [31]. These variables include important socio-demographic variables (sex, age group, province of residence, ethnicity, immigrant status, education level, income quartile), health risk variables (BMI, diabetic status, smoking status, hypertension status), and health status variables (history of selected diseases and the Health Utility Index 3). See Table 1 for an illustration of covariates in the POHEM cardiovascular disease model. These variables provide starting values for model actors’ attributes which are updated and transitioned in Step 3 (described below). The initial values of the variables are used as-is (after imputation and clean-up of the CCHS data file) or sometimes modified using an external data source. For instance, the initial estimate of diabetes prevalence based on self-reported data was modified to reflect the rate of diabetes prevalence in a disease-specific registry.

**Table 1:** Covariates and risk factors in POHEM heart disease model

CVD risk factors (# categories)	Data sources	POHEM Covariates (# categories)- Notes: SES = socioeconomic status, HS = health status, HUI = health utilities index, BMI = body mass index, CHHS = Canadian Heart Health Study, NPHS = National Population Health Survey, CHS = Canadian Health Study, CHHS = Canadian Community Health Survey, HDL = high density lipoprotein																	
		Demographics					SES		Chronic disease profile				Biophysical measures		HS	Health behaviours			
	Initial data source	5 years age groups (16)	Sex (2)	Region (5)	Ethnicity (2)	Immigrant (2)	Income (4)	Education (4)	Diabetes (2)	Heart disease (2)	Arthritis (2)	Osteoarthritis (2)	Blood pressure (5)	Total cholesterol and HDL (5)	HUI (continuous)	BMI/previous BMI (4)	Smoking (3)	Alcohol consumption (4)	Nutrition (2)
Blood pressure(5)	Imputed using CHHS (1990)	√	√						√					√		√			
Total cholesterol and HDL (5)	Imputed using CHHS (1990)	√	√						√				√			√			
Obesity (4)	NPHS (1996/97- 2004/05)	√	√	√			√	√								√			
Diabetes (2)	NPHS (1996–97)	√	√		√	√		√		√			√			√	√		
Smoking (3)	CHS (1979), NPHS (1994) and CCHS (2008)	√	√	√															

The initialization database could be replaced or supplemented with other cycles of the CCHS (or another similar survey) that contain more information about specific risk factors. For example, the CCHS does not include physical measures such as blood pressure and lipid levels. Instead, these values are imputed from the Canadian Heart Health Survey (CHHS). Together the CCHS, the CHHS, and other population-based surveys provide a suite of general and detailed health information and share more than 20 basic sociodemographic and behaviorial risk variables, which allows for imputation [32]. A disadvantage of this approach is that the extent of heterogeneity in the starting population is limited by the initial sample size and by any imputation done to prepare the file for simulation. Another disadvantage is that historical information on the duration of time with a condition or exposure is unknown (e.g., cumulative pack years smoked) and needs to be imputed.

##### **Step 3- Dynamic updates and risk transitions**

Once the start-up population is established, individuals’ disease states, risk factors, and health determinants are updated and dynamically modeled. Transitions of basic socio-demographic characteristics (age, births and deaths, immigration, and emigration and socioeconomic status) of individuals are simulated. In addition, individuals’ disease states, risk factors, and health determinants are changed dynamically by applying predictive algorithms and risk transition models. For example, in the POHEM cardiovascular disease model, the established risk factors for heart disease are updated each year for each actor from 2001 onwards. Each risk factor (including blood pressure levels, cholesterol levels, obesity, diabetes, and smoking) has an empirically-derived equation that predicts the transition of an individual from one risk factor category to another (or from one value to another, for those risk factors described continuously – e.g. BMI). See Table 1 for data sources used to derive the equations.

##### **Step 4- Validation and calibration**

Once the dynamic updates and risk transitions are applied, the estimates of the health outcomes of interest are validated. The POHEM model is validated internally (the computer code and parameter values are checked against the outputs) and where possible externally (the estimates are compared against other sources of data not used to build the model). In addition estimates are validated by specific subgroups of the population, for instance by province, age, and sex. In the case of disease incidence, such as acute myocardial infarction (AMI), POHEM projections are checked against estimates from hospital data (Canadian Institute for Health Information Discharge Abstract Database). Model output will not always agree with external benchmarks, such as cancer incidence rates from the Canadian Cancer Registry. As a result, an important step is model calibration. Calibration adjusts selected parameters in the model so that simulated estimates match observed estimates more closely.

##### **Step 5- Projection**

Once validated estimates are produced from POHEM, projected estimates of health outcomes can be examined. For example, the POHEM cardiovascular disease model currently projects estimates of heart disease risk factor prevalence and AMI hospitalizations until 2021 (the end-date is modifiable). It is important to remember that projections of cardiovascular disease risk factor prevalence and AMI hospitalizations reflect extrapolated “baseline” data, using current observed trends in risk factor prevalence and AMI hospitalizations, baseline preventive healthcare practice, and other baseline societal factors that affect cardiovascular disease risk. Baseline projections are informed by the baseline risk and health exposures of the start-up population data and the programmed risk transitions over time.

##### **Step 6- Counterfactual analysis**

**Fig. 3:**
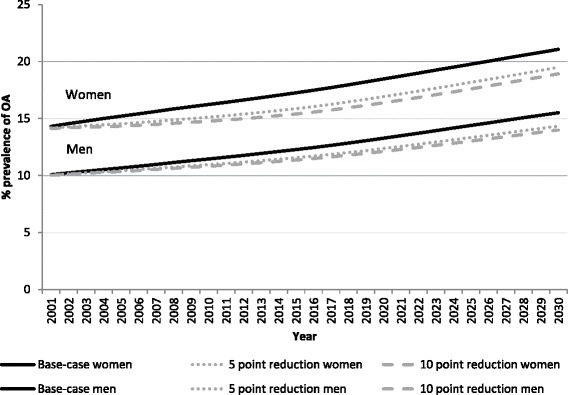
Projected osteoarthritis (OA) prevalence for overweight and obese Canadian men and women to 2031. This figure shows the prevalence of OA among overweight and obese adult Canadian men and women projected to 2031. The base-case scenario (solid black line) is contrasted with 2 other scenarios showing the projected prevalence of OA if body mass index (BMI) in 2001 was reduced by 5 points (gray dotted line) or 10 points (gray dashed line)

Once the “baseline” or “base-case” projection has been established, counterfactual analysis can begin. Depending on the outcome of interest the counterfactual scenario can result from applying a number of different interventions, from increasing the coverage of cholesterol medications in the population to reducing BMI. Figure 3 presents an illustrative counterfactual analysis of osteoarthritis (OA) prevalence for overweight and obese men and women (age 20 years and older) projected to 2031. The base-case scenario is contrasted with two other scenarios showing the projected prevalence of OA if BMI was reduced by 5 points or 10 points.

### Overview of applications

#### POHEM cardiovascular disease model

Many of the examples discussed in this paper come from the POHEM model of cardiovascular disease (see Fig. 1 and Table 1 for covariates and risk factors modeled). This model was developed in collaboration with academic research teams and has been used to investigate the projected prevalence of risk factors for heart disease in Canada [33]. POHEM was well-suited for this study because cardiovascular disease is a complex disease process, with many contributing risk factors leading to multiple outcomes. Further, the incidence of cardiovascular disease has declined in the past 50 years, with the prevalence of risk factors changing significantly. For instance, smoking is in decline while obesity is on the rise, and the population is aging. The POHEM cardiovascular disease model has projected decreased smoking rates but increased obesity prevalence, with obesity projected to overtake smoking as the most prevalent risk factor by 2017. These projections can inform policymakers interested in decreasing cardiovascular disease risk and AMI hospitalizations in the population [33].

#### POHEM cancer models

POHEM has been used extensively to study major cancer sites. Models have been developed to investigate cancer screening (colorectal and lung) [19, 23] cancer treatment (lung and breast) [15, 23] and costs of cancer (breast, colorectal, and lung) [18, 23, 34]. Flanagan *et al*. demonstrated that screening for colorectal cancer with fecal occult blood test and follow-up by colonoscopy could be cost-effective for the Canadian population but highlighted that the effectiveness of screening would greatly depend on reaching high screening participation rates [19]. These results informed the National Committee on Colorectal Cancer Screening which made updated colorectal cancer screening guidelines in Canada. Will *et al*. used the POHEM breast cancer model to demonstrate that while preventive tamoxifen treatment had a substantial benefit in reducing breast cancer incidence and mortality, the detrimental effects of tamoxifen on endometrial cancer, coronary heart disease, stroke, and deep vein thrombosis could actually outweigh its protective effects and even shorten women’s life expectancies overall [15].

The Cancer Risk Management Model (CRMM) is a spin-off model from POHEM developed by Statistics Canada in collaboration with CPAC to assess cancer control strategies in the areas of prevention, screening, and treatment for four major cancer sites: lung, colorectal, cervical and breast [23, 24]. They feature sophisticated natural history models of tumor onset and progression. Evans *et al.* projected estimates of future lung cancer incidence with current smoking rates holding steady (approximately 22 %) and counterfactually with smoking rates decreasing by 50 % over 3 years [23]. The CRMM lung module can also be used to compare the cost-effectiveness of similar smoking cessation initiatives to alternative interventions, including introduction of new screening programs, for instance, low dose CT scanning to detect early lung cancer [23, 24, 35]. The CRMM colorectal model has been used to evaluate different screening modalities (fecal occult blood, fecal immunological test, sigmoidoscopy and colonoscopy) under a range of program characteristics (age range, frequency, participation rates, costs) at a provincial level, as well as to assess the potential cost-effectiveness and budgetary impacts of implementation [36]. The CRMM cervical model can be used to evaluate the potential reorganization of cervical cancer screening in the context of vaccination for human papillomavirus (HPV) and HPV DNA testing. A wide range of scenarios have been evaluated to inform the Canadian Cervical Cancer Screening Network. This example illustrates the power of microsimulation to evaluate multiple intervention strategies including prevention, screening, and treatment, and provides common metrics with which to compare them. CRMM has also been made available on the web for easy use by cancer control and health policy analysts, who can use it to evaluate different cancer control strategies [23, 24].

#### POHEM osteoarthritis model

Osteoarthritis (OA) is the most common form of arthritis and a leading cause of disability in Canada. OA is a chronic condition of aging and as our population ages, increasing numbers of people are living with this condition and suffering reductions in their health-related quality of life. In addition, OA is costly to the healthcare system, entailing drug and surgical treatment. The POHEM model provides a tool for researchers and policymakers to examine important outcomes of OA simultaneously. Kopec *et al*. developed POHEM–OA to quantify the future health and economic burden of OA under a range of scenarios incorporating changes in a key OA risk factor (BMI) and treatment levels (medications and surgery). Simulation modeling allowed these researchers to assess the future impact of potential changes in BMI on the prevalence of OA across population groups (see Fig. 3) [20]. In addition, this model also allowed researchers to examine the effects of BMI reduction on OA incidence and health-related quality of life [37], as well as projecting the direct [38] and indirect costs of OA in the next 20 years.

#### POHEM physical activity model

POHEM-Physical Activity (PA) was developed in collaboration with PHAC to project different types of physical activity and assess the potential impact of physical activity on health outcomes, such as number of cases of disease (diabetes, hypertension, heart disease, and cancer) and health-adjusted life expectancy. The Conference Board of Canada used POHEM-PA to do counterfactual analysis of increasing physical activity among 10 % of Canadians and examined the impacts on chronic disease incidence and ultimately the Canadian economy [39]. The POHEM-PA model methodology has been formally published [22].

#### POHEM neurological model

POHEM-Neurological was developed in collaboration with PHAC to project the incidence and prevalence of neurological conditions as well as their impact on health-related quality of life, healthcare costs and caregivers [25]. The most prevalent neurological conditions were modeled: Alzheimer’s disease and other dementias, cerebral palsy, epilepsy, multiple sclerosis, Parkinson’s disease/parkinsonism, hospitalized traumatic brain injury, and hospitalized traumatic spinal cord injury. Each model accounted for changes in the Canadian population from births, immigration, emigration, and aging; however, risk factors were not included in this version of the model [25].

## Discussion

POHEM was conceived as a policy analysis tool in the early 1990s. Now, more than ever, policymakers want to use evidence-based comparative evaluations of cost and benefit when deciding to implement new medical and non-medical interventions [40]. Many of the most important health policy questions—for instance, trends in cardiovascular disease, cancer, OA, obesity, and dementia—are challenging to examine and therefore require robust and comprehensive planning models. Furthermore, Canada and other developed countries are faced with aging populations that will require more healthcare resources. Population aging and worsening health attributes, like obesity, may be contributing to a rise in chronic disease prevalence and health system costs. POHEM’s dynamic microsimulation structure is an attractive methodology to investigate complex health and health system problems.

Internationally, microsimulation and other modeling strategies have been gaining traction and have been used to inform policy in relation to complex health and health system problems. For example, in the United States a modeling collaborative funded by the National Cancer Institute, the Cancer Intervention and Surveillance Modelling Network (CISNET), has been used to inform decisions about cancer treatment and screening as well as coverage of these services by Medicaid and Medicare [11, 40]. CISNET has also contributed substantially to the development of the microsimulation modeling field by using best practices for modeling, validation, and model documentation [11, 40]. In the United Kingdom, the Foresight model has been used to investigate the future burden and costs of overweight and obesity [12]. More recent work has focused on the impact of prevention strategies on obesity related disease [13]. In Australia, Health&WealthMOD2030 has been used to project the economic impacts of early retirement from ill-health [14]. Collectively these and other simulation models have made inroads into our understanding of the health and economic impacts of cancer and chronic diseases as well as highlighting population-based prevention, treatment, and screening strategies. These models share many common attributes with POHEM. Specifically, they can be used to compare and evaluate a wide range of intervention strategies, including prevention, early detection, and therapeutic options, to assess strategies to reduce healthcare costs and improve health outcomes.

By projecting policy-relevant health outcomes, microsimulation models have the potential to allow policymakers to foresee the effects of policy. POHEM has the ability to assess multiple important health outcomes at the population level. While incidence and prevalence of disease are important health outcomes, these metrics only provide a piece of the story. Outcomes such as health-adjusted life expectancy provide a more salient picture of the full impact of disease. Projected costs of disease and treatment are another very important result, especially from a policy perspective [40]. For example, the CISNET and CRMM models can project the effect of smoking reduction on disease incidence, health resource use, and government revenues [24, 40]. In addition, microsimulation models can be useful for planning large-scale public or population health programs, such as decreasing the population’s BMI or increasing physical activity [20, 22, 39].

Implementing interventions on a large scale can be expensive and the results can be unpredictable. This methodology allows policymakers to simulate the effect of proposed interventions, and compare different interventions targeting different segments of the population [6]. The types of intervention modeled can grow beyond the health system to incorporate empirical data from any policy sphere of interest, including economic, education, and environmental policy [1]. This is an important feature if policymakers are concerned with tackling the social determinants of health and examining the distributional effects of health policies and interventions, the only caveat being that plausible scenarios can be constructed to model behaviour change as a result of interventions.

The breadth of information within population-microsimulation models like POHEM has begun to achieve the original vision of creating a comprehensive system of health statistics [1, 5]. In the United States, the Alzheimer’s Association releases an annual report with a wide range of statistics including disease projections, caregiving burden, and health care costs. POHEM-Neurological could be used to estimate almost all these measures from a single software platform, with the additional benefit of allowing counterfactual examination and sensitivity testing that will simultaneously project forward over a dozen health measures, including disease prevalence, years of life lost, healthy years of life lost, health care costs, and hours of caregiving [25].

Like all approaches, micrsosimulation has limitations. Three important limitations include intensive data requirements, difficulty quantifying and showing uncertainty estimates around model projections, and difficulty summarizing and describing the models. First, the estimates and projections produced by POHEM are only as good as the data input. Gathering, incorporating, and ensuring the quality of data is an intensive process. Ideally, data inputs reflect the Canadian population which, in practice, places a high level of reliance on population-based surveys and administrative data. Concerns with these data include sampling error, incomplete coverage, non-response, and measurement error—all of which can lead to biased results. Dynamic transitions are preferably estimated using longitudinal studies with repeated exposure and outcome measures; however, these data are uncommon. An important Canadian source of this data was Statistics Canada’s the National Population Health Survey (NPHS).

Second, it is technically challenging to quantify uncertainty estimates around model projections, yet producing such estimates is essential to establish model validity and credibility. These methods have been developed for some CISNET models and are currently under development for POHEM [41, 42]. Finally, microsimulation models can be extremely complex and have been criticised as being “black boxes.” Uptake and use of these models for population health planning and health policy evaluation requires clear communication of the methodology and assumptions used. Comprehensive model assessment, including documentation of the data sources used, quantification of the uncertainty around model projections, and clear communication of the methodology can establish model credibility and facilitate uptake by policymakers [9, 40]. Concerted efforts are being made to develop, assess, document, and use these models to answer policy-relevant questions. Specifically, the International Journal of Microsimulation, launched in 2009, has provided an avenue for researchers to publish the technical details of model development, which are of limited interest to those outside the field [43]. Further, reviews and guidelines now exist to guide researchers when constructing, validating, and reporting on microsimulation models [21, 29, 41, 44, 45].

## Conclusion

This paper has traced the origins of the POpulation HEalth Microsimulation model, sketched how it works, and provided examples of its application. Microsimulation modeling has been well established in socioeconomic policy (e.g. taxation) as well as other fields of science (e.g. environmental studies and cosmology) for many decades. Its uptake in the health field has been slow but increasing. Using POHEM as an example, this high-level overview illustrates the potential and benefits of microsimulation.

## Appendix

### Data sources

***Canadian Census of Population and Statistics Canada official population projections****—* The census is Canada’s largest and most comprehensive data source. Every 5 years the Census of Population collects demographic and linguistic information on every man, woman and child living in Canada and is the main source of data available in a standardized format for small areas. It provides nationally comparable data that can be cross-classified to show details. It is also the main body of comprehensive statistical data at the sub-provincial level on Canada’s population. The data are needed by both the public and private sectors to support decision-making in many areas, for example, to plan community services such as health care, schools, day care, police services and fire protection. Census information is used by the Demography Division at Statistics Canada to produce future projections of the numbers od new births and immigrants by age, sex and year of birth. In POHEM these data are used to initialize and update the population structure over time.

***Vital Statistics****—*Provincial and territorial Vital Statistics Acts (or equivalent legislation) render compulsory the registration of all live births, stillbirths, deaths and marriages within their jurisdictions. Under the agreement, all registrars collect a specified set of data elements, although any of them may decide to collect additional information. The central Vital Statistics Registry in each province and territory provides data from death registrations to Statistics Canada. This information is collated into statistical reports of mortality rates and causes of death. This information is used by the Demography Division, who generate the official population projections (see above), which in turn are used in POHEM.

***Canadian Community Health Survey (CCHS)****—*The CCHS is a cross-sectional survey started in 2000/01 with a sample size of 131,535. It was initially repeated every two years but as of 2009, data is collected on an ongoing basis to provide annual estimates. The CCHS was designed to be representative of the Canadian household population aged 12 years and older and elicited a wide range of self-reported information related to health status, health care utilization and health determinants. The CCHS survey and sampling strategies have been described in detail elsewhere [31]. The CCHS 2000/01 survey was used to define the initial population of the POHEM: CVD model that was projected forward in time by the simulation.

***Canadian Heart Health Survey (CHHS)****—*The CHHS are cross-sectional surveys conducted separately in each of the ten provinces between 1986 and 1992. These surveys were designed to gather information on risk factors associated with cardiovascular disease for individuals aged 18–74 years and had a combined sample size of 23,129. Unlike the CCHS, the CHHS also collected physical measures such as blood pressure and cholesterol. The CHHS survey and sampling strategies have been described in detail elsewhere [46]. In POHEM: CVD the CHHS dataset was used to initialize blood pressure and cholesterol variables and to impute values of blood pressure and cholesterol into the CCHS to allow the risk transitions for these risk factors to be updated yearly.

***National Population Health Survey (NPHS)****—*The NPHS is a longitudinal survey started in 1994/95 with a sample of 17,276 individuals aged 12 years and older. The survey is conducted every two years and currently has 18 years of follow-up. Like the CCHS, the NPHS elicited a wide range of self-reported information related to health status, health care utilization and health determinants. The NPHS survey and sampling strategies have been described in detail elsewhere [47]. In POHEM: CVD, the NPHS data set was used to model risk factors related to health behaviour and diabetes prevalence.

***Discharge Abstract Database (DAD)****—* Data on hospitalizations for AMI were obtained from the Canadian Institute of Health Information (CIHI) Discharge Abstract Database (DAD), which is a national database containing basic demographic, administrative and clinical data on hospital discharges across Canada, excluding the province of Quebec. CIHI receives data directly from participating hospitals. These include all public hospitals in every province and territory, except Quebec.

## Abbreviations

POHEM: Population health model
PHAC: Public Health Agency of Canada
CPAC: Canadian Partnership Against Cancer
CCHS: Canadian Community Health Survey
CVD: Cardiovascular disease
BMI: Body mass index
AMI: Acute myocardial infarction
OA: Osteoarthritis
CRMM: Cancer Risk Management Model
NPHS: National Population Health Survey
CCORT: Canadian cardiovascular outcomes research team
STAR: Simulation technology for applied research team
